# GFP tagging of *Brucella melitensis* Rev1 allows the identification of vaccinated sheep

**DOI:** 10.1111/tbed.13053

**Published:** 2018-11-26

**Authors:** Ana Zabalza‐Baranguá, Beatriz San‐Román, Carlos Chacón‐Díaz, María‐Jesús de Miguel, Pilar‐María Muñoz, Maite Iriarte, José‐María Blasco, María‐Jesús Grilló

**Affiliations:** ^1^ Instituto de Agrobiotecnología (IdAB, CSIC‐Gobierno de Navarra) Mutilva, Navarra Spain; ^2^ Centro de Investigación en Enfermedades Tropicales Facultad de Microbiología Universidad de Costa Rica San José Costa Rica; ^3^ Centro de Investigación y Tecnología Agroalimentaria (CITA) Instituto Agroalimentario de Aragón (IA2) Gobierno de Aragón Zaragoza Spain; ^4^ Instituto de Salud Tropical – Dpto. de Microbiología y Parasitología Universidad de Navarra Pamplona Spain

**Keywords:** *Brucella melitensis*, ELISA‐GFP, mini‐Tn*7*‐*gfp*, sheep, vaccines

## Abstract

Brucellosis is a worldwide zoonosis causing important economic loss and a public health problem. Small ruminants are the preferred hosts of *Brucella melitensis* and thus the main source of human infections. Effective control of sheep and goat brucellosis has been achieved in several countries through vaccination with the live‐attenuated *B. melitensis* Rev1 vaccine. However, Rev1 induces a long‐lasting serological response that hinders the differentiation between infected and vaccinated animals. A Rev1::*gfp* strain expressing constitutively the Green Fluorescent Protein (GFP) was built by stable insertion of a mini‐Tn*7*‐*gfp* in the *glmS‐recG* non‐codifying chromosomal region. An associated indirect ELISA‐GFP was developed to identify anti‐GFP antibodies in vaccinated animals. The resulting Rev1::*gfp* kept the biological properties of the Rev1 reference strain, including residual virulence and efficacy in mice, and was readily distinguished from Rev1 and other *Brucella* field strains by direct visualization under ultraviolet illumination, fluorescence microscopy and a multiplex PCR‐GFP. The Rev1::*gfp* strain did not elicit anti‐GFP antibodies itself in lambs but when applied in combination with recombinant GFP induced an intense and long‐lasting (>9 months) anti‐GFP serological response readily detectable by the ELISA‐GFP. Overall, our results confirm that Rev1 GFP‐tagging can be a suitable alternative for identifying vaccinated sheep in infected contexts.

## 1 INTRODUCTION

Brucellosis is a widespread disease caused by *Brucella* species that affects a large variety of domestic and wildlife hosts, including humans (Moreno & Moriyón, 2006). *Brucella melitensis* is the main agent of human brucellosis, which is related directly to infection in sheep and goats. The disease induces abortions and reproductive disorders in small ruminants, causing important economic and public health problems (Corbel, 2006; Dean, Crump, Greter, Schelling, & Zinsstag, 2012; Zinsstag, Schelling, Solera, Blasco, & Moriyón, 2011). Due to the absence of suitable vaccines for humans, prevention depends on the control/eradication of the disease in animals. Vaccination, combined or not with test & slaughter, is the only practical strategy to control this important zoonosis in most affected areas (Blasco, 1997; Blasco & Molina‐Flores, 2011). *B. melitensis* Rev1 is the only vaccine available against sheep and goat brucellosis (OIE, 2016). The protective efficacy of this live vaccine is based on its residual virulence, a critical property required to induce a long‐lasting effective immunity against field infections (Bosseray & Plommet, 1990). However, Rev1 vaccination induces anti‐smooth lipopolysaccharide (S‐LPS) antibodies that are indistinguishable of those generated after field *Brucella* infections, leading to interferences in conventional anti‐LPS tests (Ducrotoy, Conde‐Álvarez, Blasco, & Moriyón, 2016). Vaccination of exclusively young replacements minimizes this interference (particularly when Rev1 is applied by the conjunctival route) but does not fully solve the problem of differentiating vaccinated animals (Fensterbank, Pardon, & Marly, 1982, 1985).

Several approaches to generate vaccines or strategies to allow differentiation between infected and vaccinated animals (DIVA) have been studied (Blasco, Moreno, & Moriyón, 2016; Yang et al., 2013). Since most infected animals react against *Brucella* cell envelope antigens, efforts have been focused on the removal of immunogenic relevant proteins such as outer membrane and binding proteins (Cloeckaert et al., 2004; Grilló et al., 2009; Guilloteau et al., 2006; Jacques et al., 2007) or O‐polysaccharide epitopes (Godfroid et al., 2000; González et al., 2008). Unfortunately, these approaches entail eventual inconveniences that limit the practical usefulness of the tagging deletion such as: (a) an excessive attenuation and/or loss of vaccine efficacy (Barrio et al., 2009; González et al., 2008); (b) the lack of specificity of the associated tests to detect specific antibodies against the deleted antigen in infected animals (Grilló et al., 2009); and (c) a potential positive selective advantage for virulent bacteria against the attenuated vaccine strain (Moreno, 2014).

An alternative approach has been the inclusion of antigens xenogenic for *Brucella*, such as *Trypanosoma cruzi* immunogenic proteins (Comerci, Pollevick, Vigliocco, Frasch, & Ugalde, 1998; Pollevick, Affranchino, Frasch, & Sanchez, 1991) or the Green Fluorescent Protein (GFP) from the *Aequorea victoria* jellyfish (Chacón‐Díaz et al., 2011) in combination with associated diagnostic tests. Both approaches were performed by encoding the xenogenic antigens through a non‐integrative plasmid, limiting the usefulness of these vaccines in field conditions.

We have developed a Rev1 vaccine strain carrying the *gfp* gene stably inserted in the chromosome (Rev1::*gfp*) that maintains the biological properties of the reference vaccine and is readily identified by ultraviolet (UV) illumination and PCR‐GFP multiplex. In this work, several immunization strategies were conducted in mice and then in lambs to induce anti‐GFP antibodies identified by an associated indirect ELISA‐GFP, thus, allowing the identification of vaccinated animals.

## 2 MATERIAL AND METHODS

### 2.1 Bacterial strains, growth conditions, inocula preparation, plasmids used and DNA manipulations

The bacterial strains and plasmids used in this study are listed in Table 1. Bacteria were routinely grown in Blood Agar Base n°2 (BAB; Oxoid), Luria Bertani Broth (LB; Pronadisa) or Trypticase Soy Broth (TSB; Pronadisa) either plain or supplemented with selected antibiotics (all from Sigma‐Aldrich Química) such as kanamycin 50 μg/ml (Km_50_) or 35 μg/ml (Km_35_), ampicillin 100 μg/ml (Amp_100_), polymyxin B 1.5 μg/ml (PxB_1.5_), gentamicin 15 μg/ml (Gm_15_), streptomycin 2.5 μg/ml (Str_2.5_) or penicillin G 5 μg/ml (P_5_). Bacterial strains were stored at −20°C in 10% skimmed milk supplemented with 3% sterile lactose (Applichem Panreac).

**Table 1:** Bacterial strains and plasmids used in this work

*Brucella melitensis*	Properties and use in this work	Source/References
Rev1	*B. melitensis* Rev1 vaccine reference strain	IdAB collection
Rev1::*gfp*	Rev1 carrying the mini‐Tn*7*‐*gfp* inserted into the *att*Tn*7* site in the intergenic *glmS*‐*recG* chromosomal region*;* Km_50_^r^	This work
Rev1pGFP	Rev1 carrying the non‐integrative plasmid pBBR1‐2‐*gfp*‐Km; Km_50_^r^; used as control of high *gfp* expression	IdAB collection
H38::Gm	*B. melitensis* biovar 1 carrying mini‐Tn*7*‐*gfp*‐Gm; Gm_15_^r^; used in challenge experiments in mice	IdAB collection

*Escherichia coli*/plasmid		
S17 (λpir)/pUC18T‐mini‐Tn*7*‐*gfp*‐Gm	*E. coli* S17 (λpir) carrying the pUC18T‐mini‐Tn*7*T‐Gm‐*rrnB* P1::*gfpmut3*T0T1 (http://www.ncbi.nlm.nih.gov/pubmed; accession no. DQ493877.2; plasmid named “pUC18T‐mini‐Tn7T‐Gm‐*gfpmut3*”); Amp_100_^r^; Gm_15_^r^; used for extracting the *rrnB* P1‐*gfp* fragment.	(Choi & Schweizer, 2006)
TOP10F´/pCR2.1‐*gfp*	*E. coli* TOP10F’ carrying the pCR2.1‐TOPO TA Cloning kit (Thermo Fisher Scientific) containing *rrnB* P1‐*gfp*; Km_50_^r^.	This work
pUC18R6KT‐mini‐Tn*7*‐Km	pUC18R6KT‐mini‐Tn*7*TKm; Amp_100_^r^ and Km_50_^r^; used for cloning the *rrnB* P1‐*gfp* from pCR2.1‐*gfp*.	(Llobet et al., 2009)
S17 (λpir)/pUC18R6KT‐mini‐Tn*7*‐*gfp*‐Km	*E. coli* S17 (λpir) carrying the pUC18R6KT‐mini‐Tn*7*‐Km‐*rrnB* P1::*gfpmut3*T0T1 suicide plasmid; Amp_100_^r^ and Km_50_^r^; used to insert the *rrnB* P1‐*gfpmut3* in the intergenic region between *glmS* and *recG* of the *Brucella* chromosome.	This work
SM10 (λpir)/pTNS2	*E. coli* SM10 (λpir) carrying the plasmid pTNS2 with TnsABCD transposase genes, required for the insertion and orientation of Tn*7*; Amp_100_^r^	(Choi & Schweizer, 2006)
HB101/pRK2013	*E. coli* carrying the helper plasmid pRK2013; Km_35_^r^; necessary for the mobilization of plasmids involved in the mini‐Tn*7* insertion by conjugation.	(Choi & Schweizer, 2006)
XL1‐Blue/pGEX‐4T‐1‐*gfp*	*E. coli* GFP‐GST expression system; Amp_100_^r^; necessary for obtaining recombinant GFP.	(Chacón‐Díaz et al., 2011)

Km: kanamycin; Gm: gentamicin; Amp: ampicillin; ^r^: resistant.

*Brucella* suspensions were prepared in sterile Phosphate Buffered Saline (PBS; pH 7.2) by spectrophotometry (SmartSpec Plus Spectrophotometer, BioRad) and exact doses were determined retrospectively, as described previously (Grilló et al., 2006). Also, inocula of purified recombinant GFP (see below) were prepared in PBS, sterilized through 0.22 μm filters (Millipore^®^) and further quantified by Bradford (BioRad).

*Escherichia coli* S17 (λpir) strains carrying specific plasmids for mini‐Tn*7* based integration assays, that is, pUC18T‐mini‐Tn*7*‐*gfp*‐Gm (Choi & Schweizer, 2006) and pUC18R6KT‐mini‐Tn*7*‐Km (Llobet, March, Giménez, & Bengoechea, 2009), were kindly provided by Prof. Herbert P. Schweizer (Colorado State University, Fort Collins, Colorado, USA) and Prof. José Bengoechea (School of Medicine, Dentistry and Biomedical Sciences, Queen's University Belfast, Ireland), respectively.

All primers were synthesized in Sigma‐Aldrich Química SL (Madrid, Spain). Plasmid and chromosomal DNA were extracted with Qiaprep Spin Miniprep^®^ (Qiagen GmbH) and Ultraclean^®^ Microbial DNA Isolation Kit (Mo Bio Laboratories), respectively. Single‐colony DNA extraction was performed by boiling (100°C, 20 min) in 100 μl of sterile ultrapure water, followed by centrifugation at 1,073 × *g*, for 10 min.

### 2.2 Construction of pUC18R6KT‐mini‐Tn*7*‐*gfp*‐Km

The plasmid pUC18T‐mini‐Tn*7*‐*gfp*‐Gm was used to generate the cloning vector pCR2.1‐*gfp* carrying both the *gfp* gene and the *E. coli rrnB* P1 ribosomal promoter (Table 1) by: (a) *gfp* amplification from the pUC18T‐mini‐Tn*7*‐*gfp*‐Gm by PCR using the primers rrnBP1‐F and Gfp_f‐R2 (Table 2); (b) extraction of a DNA fragment (ATP Gel/PCR Extraction Kit, ATP biotech Inc.) from the gel agarose electrophoresis; and (c) insertion of the DNA fragment into the pCR2.1‐TOPO (TOPO TA Cloning kit, Thermo Fisher Scientific). Then, the pUC18R6KT‐mini‐Tn*7*‐*gfp*‐Km (Table 1) was constructed by digestion of the pCR2.1‐*gfp* with EcoRI (Takara) for extracting the *rrnB* P1‐*gfp* fragment and further insertion into pUC18R6KT‐mini‐Tn*7*‐Km by using a T4 ligase (Invitrogen). The resulting pUC18R6KT‐mini‐Tn*7*‐*gfp*‐Km was checked by sequencing (Secugen) and, then, transformed in *E. coli* S17 (λpir) by thermal shocking.

**Table 2:** Oligonucleotides used for the construction and identification of Rev1::*gfp* vaccine

Primers	Hybridizing region	Size of DNA amplification in Rev1::*gfp*	Sequence (5′ → 3′)
rrnBP1‐F	*E. coli rrnB* P1 promoter nucleotides 1–25 (forward)	816 bp	GTTGCGCGGTCAGAAAATTATTTTA
Gfp_f‐R2	*gfp* 232‐stop codons (reverse)		TTATTTGTATAGTTCATCCATGCCA
GlmS_B^a^	*glmS* internal region codons 588–595 (forward)	200 bp	GTCCTTATGGGAACGGACGT
Tn7‐R^a^	mini‐Tn*7* upstream (reverse)		CACAGCATAACTGGACTGATT
Gfp‐F^a^	*gfpmut3* internal region codons 65–70 (forward)	432 bp	TCGGTTATGGTGTTCAATGC
Gfp‐R^a^	*gfpmut3* internal region codons 202–208 (reverse)		AAAGGGCAGATTGTGTGGAC
Tn7‐L	mini‐Tn*7* downstream (forward)	350 bp	ATTAGCTTACGACGCTACACCC
RecG^a^	*recG* internal region codons 671–678 (reverse)		TATATTCTGGCGAGCGATCC

a Primers used in PCR‐GFP multiplex for Rev1::*gfp* identification (Figure 1). In Rev1 and other conventional *Brucella*, GlmS_B and RecG primers amplify a 328 bp band.

### 2.3 Construction and genetic characterization of Rev1::*gfp*

The chromosomal insertion of the *gfp* in *B. melitensis* Rev1 was performed by the mini‐Tn*7* directed mutagenesis method, using the suicide vector pUC18R6KT‐mini‐Tn*7*‐*gfp*‐Km. This method allows the *gfp* insertion into the *att*Tn*7* non‐codifying site usually located 25 nucleotides downstream of the glucosamine‐fructose‐6‐phosphate aminotransferase (*glmS*) gene, which is highly conserved amongst bacteria and present in *Brucella* (Choi & Kim, 2009; Choi & Schweizer, 2006; Choi et al., 2005). Thus, a preliminary study in silico was performed in order to assess the exact chromosomal localization and the percentage of homology of *glmS* in *B. melitensis* with respect to other brucellae. For this, the Blast‐Tools of the National Center for Biotechnology Information (NCBI) database (NCBI, 2018) for *Brucella* spp. and sequencing (Secugen) of the *glmS* downstream *att*Tn*7* site were performed in Rev1 by using the GlmS_B and RecG primers (Table 2). Thereafter, the protocol of mini‐Tn*7*‐*gfp* insertion between *glmS* and *recG* genes described by Choi (Choi & Schweizer, 2006) was adapted to *B. melitensis* Rev1 strain (Figure 1a). Briefly, a tetraparental conjugation was achieved by mixing 0.5 ml of the Rev1 receptor strain previously grown in TSB (37°C, overnight) and 0.2 ml of each *E. coli* S17 (λpir)‐pUC18R6KT‐mini‐Tn*7*‐*gfp‐*Km, *E. coli* SM10 (λpir)‐pTNS2 and *E. coli* HB101‐pRK2013, all previously grown (37°C, overnight) in LB supplemented with the corresponding antibiotic of selection (Table 1). The bacterial mix was washed twice with 1.5 ml of MgSO_4_ 10 mM, dissolved in 30 μl of MgSO_4_ and dropwise cultured (37°C, 6 hr) in BAB. Finally, bacteria were harvested in PBS, decimally diluted and cultured (37°C, 5–6 days) in a BAB‐PxB_1.5_‐Km_50_ plate to select the desired Rev1::*gfp* transformed clones.

**Figure 1:**
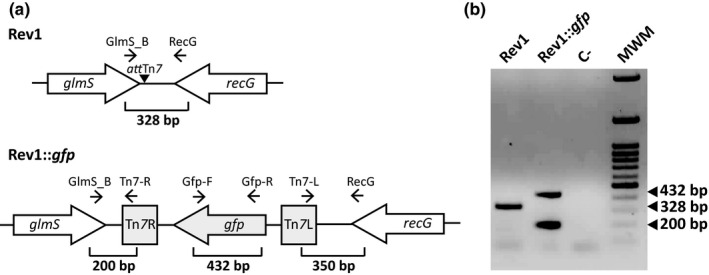
Genetic characterization of mini‐Tn*7‐gfp* chromosomal insertion in Rev1::*gfp*. (a) Schematic representation of *glmS‐recG* region of *Brucella melitensis* Rev1 (upper panel) and integration of the *gfp* gene in Rev1 by mini‐Tn*7*‐*gfp* insertion in the *att*Tn*7* site of the *glmS‐recG* region (lower panel). (b) Molecular identification of Rev1::*gfp* by multiplex PCR‐GFP with the GlmS_B, RecG, Tn7‐R, Gfp‐F and Gfp‐R primers allowing differentiation between mini‐Tn*7*‐*gfp* tagged vaccines (200 and 432 bp) and Rev1 or other *Brucella* conventional strain (328 bp). C‐: control negative (water); and MWM: Molecular Weight Marker

The proper insertion and orientation of the mini‐Tn*7*‐*gfp* were assessed in ten Rev1::*gfp* transconjugant clones by using four individual PCR with the following pairs of primers: (a) GlmS_B and Tn7‐R; (b) Tn7‐L and RecG; (c) Gfp‐F and Gfp_R; and (d) GlmS_B and RecG, as described in Table 2. As represented in Figure 1a, these four PCR amplified, respectively, DNA fragments of: (a) 200 bp from the mini‐Tn*7* insertion downstream the *glmS* gene; (b) 350 bp from the mini‐Tn*7* insertion upstream the *recG* gene; (c) 432 bp of the *gfpmut3* gene inserted in the mini‐Tn*7*; and (d) 328 bp of the wild type intergenic region between *glmS* and *recG* genes, that is, negative amplification in Rev1::*gfp* transconjugants.

### 2.4 Phenotypic characterization of Rev1::*gfp*

#### 2.4.1 Classical microbiological markers

Three clones of Rev1::*gfp* showing the desired genetic characteristics were selected for further phenotypic characterization. Colonial size, crystal violet‐oxalate exclusion, catalase, oxidase, urease and acriflavine tests (all products from Sigma Aldrich, Spain), sensitivity to Tb, Wb, Iz and R/C phages, agglutination with anti‐A and anti‐M monospecific sera, both CO_2_‐ and serum‐ dependence, susceptibility to both dyes (i.e., thionine blue 10, 20 and 40 μg/ml, fuchsine 10 and 20 μg/ml, and safranin 100 μg/ml; Sigma) and antibiotics (i.e., penicillin and streptomycin) markers were assessed by standard protocols (Alton, Jones, Angus, & Verger, 1988).

#### 2.4.2 In vitro growth

Bacterial growth curves were determined in vitro by both spectrophotometry and bacterial Colony Forming Units (CFU) counts. Briefly, TSB suspensions containing 1 × 10^2^ CFU/ml were incubated (37°C, under shaking) for 7 days, and tested at selected intervals for assessing the Optical Density at 600 nm (OD_600 nm_) and the CFU/ml on BAB plates. The mean ± *SD* (*n* = 3) values of individual OD_600 nm_ readings and log_10_ CFU/ml were calculated and compared statistically by a one‐way ANOVA followed by the Fisher's Protected Least Significant Differences (PLSD) test.

#### 2.4.3 Stability of GFP tagging in Rev1::*gfp*

The stability of the *gfp* insertion and GFP expression in Rev1::*gfp* was assessed after: (a) 20 subcultures in BAB plates; (b) storage of cultures in plates at 4°C for 3 months; (c) two consecutive passages in mice (Grilló, Blasco, Gorvel, Moriyón, & Moreno, 2012); and (d) each spleen culture in mice virulence experiments. DNA from a representative number of individual colonies from each subculture or spleen culture were extracted and checked by a one‐step multiplex PCR‐GFP using a mix of GlmS_B, Tn7‐R, RecG, Gfp‐F and Gfp‐R primers (Table 2) which allows the amplification of either a double band of 200 bp and 432 bp in Rev1::*gfp* or a single band of 328 bp in conventional *Brucella* spp. (Figure 1).

The GFP expression in Rev1::*gfp* was assessed by direct identification of bacterial colonies grown in BAB plates in an UV transilluminator (Ultralum, Claremont, USA) at 365 nm, and by epifluorescence microscopy at 457.5–492.5 nm excitation (FITC filter; Nikon 80i). Moreover, expression and semi‐quantification of the GFP produced by Rev1::*gfp* was estimated by Western‐Blot (WB) using 15 μl/lane of each bacterial lysate containing 1 × 10^9^ CFU/ml and a monoclonal mouse anti‐GFP serum (Clontech; 1/5,000). Rev1 and Rev1pGFP (Table 1) lysates were used as negative and positive controls, respectively, and a gradient of recombinant GFP containing 6, 3, 1.5 or 0.75 μg/ml of GFP was used as protein ladder. The pre‐stained Protein Marker VI (AppliChem) was used as molecular marker of 10–245 kDa.

### 2.5 Recombinant GFP production, characterization and study of stability

Recombinant GFP labelled with glutathione‐S‐transferase (GST) was obtained and purified by affinity chromatography (GST GraviTrap^®^ Columns Gravity Flow Purification; GE Healthcare^®^) from the soluble fraction of *E. coli* XL1‐Blue harbouring the plasmid pGEX‐4T‐1‐*gfp* (Table 1). After purification, GFP was excised from the fusion protein using an enzymatic treatment (37°C, 24 hr) with 100 IU/mg thrombin (GE Healthcare^®^). The purity and antigenicity of both GFP isoforms were determined by SDS‐PAGE followed by Coomassie staining and WB anti‐GFP, as described above. The stability to temperature was assessed by incubating GFP‐GST and GFP suspensions (100 μl aliquots) at 4, 37, 44 and 60°C, for 9 weeks. Moreover, the stability to UV radiation was assessed by submitting the GFP suspensions placed in 96‐well plates (60 μl/well; Maxisorp, Nunc^®^) to an UV intensity of 11.34 J, administered in 18 pulses (i.e., equivalent to 90 hr of natural outdoor exposure) in a microwave irradiator (Stratalinker). In both experiments, the GFP stability was determined by both direct visualization of fluorescence under UV illumination and WB antigenicity procedures, as described above.

### 2.6 ELISA‐GFP validation

The presence of anti‐GFP antibodies was determined by an indirect ELISA‐GFP in both mice and sheep studies (see below). For this, 96 well plates (Maxisorp, Nunc^®^) were coated with 1 μg/well of GFP in 0.1 M carbonate buffer, blocked with skimmed milk (2% in PBSTween 0.1%) and tested with 1/100 serum dilutions in PBS. Reactions were revealed using the corresponding peroxidase conjugate (i.e., anti‐IgG H+L rabbit anti‐mouse at 1/5,000 in PBS or Protein G diluted at 1/4,000 in PBS, for mice and sheep, respectively; both conjugates were from Thermo Scientific) and ABTS (Millipore) as substrate. After 30 min incubation, Optical Density readings with a 405 nm filter (OD_405 nm_) were assessed in a Multiscan microplate reader (Labsystem). As controls in each plate, one GFP‐negative serum and one GFP‐positive serum from hyperimmunized mouse/lamb (see Sections 2.7.3 and 2.8.1) were used. The results were expressed either as % OD_405 nm_ = [(A/C_positive_) × 100] for determining the cut‐off and the percentage of positive animals in the ELISA‐GFP; or as OD_405 nm_ index = (A–C_negative_) for determining the level of positivity of the sera analyzed. In both formulas, A is the raw data of absorbance obtained for the sample tested; and C_positive_ and C_negative_ are, respectively, the absorbance values of the positive and negative controls of each plate.

The ELISA‐GFP cut‐off and associated sensitivity (Se) and specificity (Sp) for mice and sheep were established using a selection of gold standard sera from GFP‐negative (30 from mice and 124 from sheep) and GFP‐positive (36 from mice and 47 from sheep) animals. The GFP‐positive sera were obtained by blood extractions at different point‐times in the hyper‐immunization assays (see Sections 2.7.3 and 2.8.1) and subsequent dilutions in PBS in order to check different levels of positivity. The GFP‐negative sera were obtained from GFP‐non‐immunized mice (CSIC‐IdAB collection) and sheep (CITA collection), being the latter from 77 *Brucella*‐free and 47 *B. melitensis*‐infected animals. The % OD_405 nm_ values obtained by duplicate with the gold standard sera were processed by the 2016 MEDCALC^®^ Statistical Software bvba version 16.8.4 (MEDCALC, 2016) and the cut‐off selected was that resulting in 100% Se and Sp for discriminating both GFP‐positive and GFP‐negative populations.

### 2.7 Mice studies

Eight‐week old BALB/c female mice (Charles River) were accommodated for 1 week in the P3 facilities (registration code ES/31‐2016‐000002‐CR‐SU‐US) of the IdAB (Navarra, Spain). Animals were kept in cages with water and food ad libitum under biosafety containment conditions. Animal handling and procedures were in accordance with the current Spanish (RD 53/2013) and European (Directive 14/86/609/EEC) legislations, and following the FELASA (Rehbinder et al., 2000) and ARRIVE (Kilkenny, Browne, Cuthill, Emerson, & Altman, 2010) international recommendations.

General procedures in mice were performed as described previously (Grilló et al., 2006, 2012). Briefly, bacterial inocula were prepared in sterile PBS just before administration, so that each mouse received the inoculation dose in 0.1 ml, either intraperitoneally (IP) or subcutaneously (SC). The exact doses of *Brucella* were determined retrospectively as described elsewhere (Grilló et al., 2006). Blood samples were taken by retrorbital plexus puncture and the serum samples were obtained by centrifugation (4,000 rpm, 10 min) and kept at −20°C until use. At the end of each experiment, mice were slaughtered by cervical dislocation and the spleens were removed and processed individually, in sterile conditions, for determining the mean ± *SD* (*n* = 5) of individual log_10_ CFU/spleen, at each selected point‐time (Grilló et al., 2012). Statistical comparisons of means were performed by one‐way ANOVA and the Fisher's PLSD tests.

#### 2.7.1 Virulence

Groups of 25 mice were inoculated IP with 1 × 10^6^ CFU/mouse of Rev1::*gfp* or Rev1 reference strain (control) and the number of log_10_ CFU/spleen was determined at 1, 3, 6, 9 and 12 weeks after inoculation, as described elsewhere (Grilló et al., 2012).

#### 2.7.2 Vaccine efficacy

The protection conferred by Rev1::*gfp* against a virulent challenge in mice was assessed following standard procedures (Grilló et al., 2012; OIE, 2016). Briefly, groups of 10 mice were immunized SC with 2 × 10^5^ CFU/mouse of Rev1::*gfp* or Rev1 reference type (vaccine control) or with sterile PBS (unvaccinated control). Four weeks later, all mice were challenged IP with 1×10^4^ CFU/mouse of *B. melitensis* H38::Gm virulent strain (Table 1) and, 2 weeks after challenge, the log_10_ CFU/spleen of the virulent strain was determined by plating in BAB‐Gm_15_ (Grilló et al., 2006).

#### 2.7.3 Obtaining GFP‐hyperimmunized sera

BALB/c mice (*n* = 5) were inoculated SC three consecutive times, (at day 0, week 4 and week 6) with 0.1 ml of a suspension containing 20 μg of recombinant GFP mixed 1:1 (vol:vol) with Incomplete Freund's Adjuvant (IFA, Sigma‐Aldrich, Spain). Pre‐immunization sera were obtained and used as GFP‐negative controls of the ELISA‐GFP plates (see Section 2.6).

#### 2.7.4 Serological response after vaccination

Two consecutive experiments were performed:

In the first experiment we assessed the anti‐GFP response (OD_405 nm_ index) in mice vaccinated with Rev1::*gfp*, and the effect of the combined administration with recombinant GFP. Five groups (*n* = 5) of BALB/c mice were inoculated with: (a) Rev1::*gfp*; (b) Rev1::*gfp* mixed 1:1 with recombinant GFP; (c) Rev1 reference strain mixed 1:1 with recombinant GFP; (d) Rev1 reference strain alone; and (e) recombinant GFP alone. Each mouse received IP 0.1 ml of the correspondent suspension containing 1 × 10^6^ CFU of Rev1::*gfp* or Rev1 and/or 20 μg of recombinant GFP in sterile PBS. Blood samples were taken just before immunization and at week 6 post‐immunization.

In the second experiment we determined the evolution of the percentage of mice positive in ELISA‐GFP after vaccination and the effect of a booster with recombinant GFP. Ten mice were immunized IP with Rev1::*gfp* mixed 1:1 with recombinant GFP, as in the first experiment, and 14 weeks thereafter all mice received a SC booster with recombinant GFP (20 μg/mouse) either in PBS (*n* = 5) or mixed with 6 μg of aluminum hydroxide (AlH; CZ Veterinaria S.A., Spain) (*n* = 5) as adjuvant (Shaw, Li, & Tomljenovic, 2013). A group of five mice inoculated IP with 1 × 10^6^ CFU of Rev1 was used as control. Blood samples were taken just before immunization and then weekly or fortnightly until 36 weeks post‐immunization.

### 2.8 Sheep studies

A total of 14 male and 14 female 4–5 months old Rasa Aragonesa lambs born in the *Brucella*‐free flock of the CITA de Aragón (Spain) were used for assessing the safety and serological response induced by Rev1::*gfp*. Lambs were kept in the animal facilities of the CITA (registration code ES/50‐2970‐12005) during the study and handled by qualified personnel following the FELASA (Rehbinder et al., 2000) and ARRIVE (Kilkenny et al., 2010) international welfare recommendations.

After SC immunizations, clinical monitorization was performed to study systemic or local reactions, by measuring rectal temperature at 0, 1, 2, 3 and 7 days post‐inoculation and by palpation and examination of the point of inoculation. Blood samples were taken by jugular vein punction with Venojet^®^ tubes (Terumo) and the sera obtained by centrifugation (2,500 × *g*, 10 min) were kept at −20°C until analysis. All sera were tested for anti‐S‐LPS antibodies by the standard Rose Bengal (RBT) and Complement Fixation (CFT) tests performed by the standard procedures (OIE, 2016), and for anti‐GFP antibodies by ELISA‐GFP (see Section 2.6).

#### 2.8.1 Obtaining GFP‐hyperimmunized sera

Lambs (*n* = 6) were inoculated SC three consecutive times, (at day 0, week 4 and week 6) with 0.5 ml of a suspension containing 62.5 μg of recombinant GFP mixed 1:1 (vol:vol) with IFA. Pre‐immunization sera were obtained and used as GFP‐negative controls of the ELISA‐GFP plates.

#### 2.8.2 Serological response after vaccination

Based on the results obtained in mice, two sequential experiments were designed to study the serological responses after vaccination in lambs.

The first experiment was conducted to assess (a) the ability of Rev1::*gfp* to generate anti‐GFP antibodies; (b) the enhancement of anti‐GFP response induced when Rev1::*gfp* was combined with recombinant GFP; and (c) the effect in the anti‐GFP response when the combination Rev1::*gfp*+GFP was complemented with a booster with recombinant GFP in either PBS or IFA adjuvant. For this, lambs were vaccinated SC either (*n* = 6) with 1 ml of a suspension containing 1–2 × 10^9^ CFU of Rev1::*gfp* prepared as described (Barrio et al., 2009) or (*n* = 8) with 2 ml of a suspension containing 1 ml of Rev1::*gfp* and 1 ml of a sterile suspension with 100 μg recombinant GFP. Serum samples obtained at week 3 post‐vaccination were tested by the ELISA‐GFP and expressed as OD_405 nm_ index. Six weeks after vaccination, when a significant decrease of the anti‐GFP response was observed in most of animals, the latter group was boosted SC with 100 μg/lamb of recombinant GFP diluted either in PBS (*n* = 4) or IFA adjuvant (*n* = 4). The evolution of the percentage of reactors in ELISA‐GFP was graphically represented. Safety of vaccination and serological responses induced anti‐S‐LPS and anti‐GFP was monitored at regular intervals until week 39 after vaccination.

The second experiment was designed to compare the behaviour of both anti‐GFP and anti‐S‐LPS responses under the best conditions defined in the first experiment. A group of eight lambs was vaccinated SC each with 1–2 × 10^9^ CFU of Rev1::*gfp* mixed 1:1 (vol:vol) with 100 μg of recombinant GFP in PBS. Ten weeks later, when most lambs showed undetectable anti‐GFP antibodies, a booster (2 ml, SC) with 100 μg of recombinant GFP mixed with 6 mg of AlH adjuvant was administered to each lamb. Safety of vaccination and serological responses (% of reactors) induced anti‐S‐LPS and anti‐GFP was monitored at regular intervals until week 36 after vaccination.

## 3 RESULTS

### 3.1 Rev1::*gfp* carried the mini‐Tn*7*‐*gfp* inserted between *glmS* and *recG* genes of *B. melitensis* Rev1

The in silico analysis showed only one *glmS* gene in brucellae that is highly conserved amongst various species (99% homology; Supporting information Table S1). Rev1 *glmS* was not annotated in NCBI database. DNA sequencing confirmed the presence of *att*Tn*7* site and the Tn*7*‐*gfp* insertion at 25 nucleotides downstream of the *glmS* gene in Rev1::*gfp*, as reported for other bacterial species (Choi & Schweizer, 2006). The ten Rev1::*gfp* clones checked by different PCR showed the expected genotype (Table 2, Figure 1).

### 3.2 Rev1::*gfp* kept the phenotype of the *B. melitensis* Rev1 reference strain and expressed GFP

**Figure 2:**
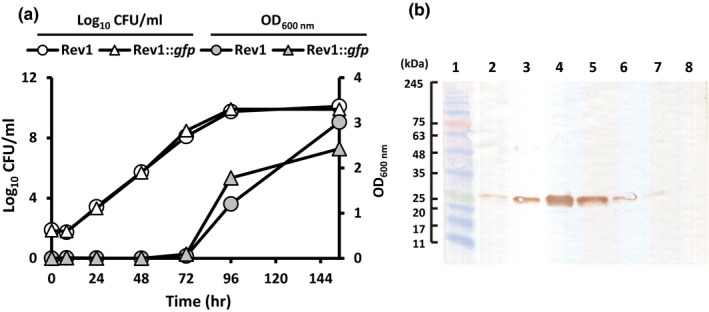
Rev1::*gfp* displayed the in vitro properties of the parental Rev1 strain and expressed GFP stably. (a) In vitro growth kinetics of Rev1::*gfp* (triangles) or Rev1 (circles) during 154 hr of incubation in TSB medium, determined by both log_10_ CFU/ml counts (white symbols, left axis) and absorbance reads at OD600 nm (grey symbols, right axis)_._ Values represent the mean ± *SD* of triplicate measures. (b) Relative estimation of GFP expression in Rev1::*gfp* (15 μl of a 1 × 10^9^ CFU/ml of inactivated bacteria; Line 2) by Western‐blot anti‐GFP with respect to a gradient (from 6 to 0.75 μg/ml; Lines 4–7) of purified GFP processed in well‐standardized conditions. Rev1‐pGFP (Line 3) and Rev1 (Line 8) were used as positive and negative controls, respectively. Line 1: molecular weight marker [Colour figure can be viewed at wileyonlinelibrary.com]

Rev1::*gfp* showed the phenotypic characteristics of its isogenic Rev1 parental strain (Grilló, Bosseray, & Blasco, 2000), in particular those regarding: (a) small colony size (1–1.2 mm diameter) after 5 days of incubation at 37°C; (b) smooth appearance after crystal violet‐oxalate staining (in ≈5,000 CFU analyzed) and acriflavine tests; (c) growth on BAB‐Str_2.5_ and inhibition on BAB‐P_5_ plates; and (d) the in vitro growth kinetics (Figure 2a). Also, Rev1::*gfp* emitted fluorescence detectable by both direct UV illumination and epifluorescence microscopy allowing its straightforward distinction from both the parental Rev1 and field *B. melitensis* strains (not shown). The Rev1::*gfp* phenotype was stable, since all colonies recovered after serial subcultures or passages in mice kept the fluorescent phenotype and the *gfp* gene inserted at the *att*Tn*7* site. Estimation by WB indicated that 7 × 10^6^ Rev1::*gfp* CFU produced between 0.75 and 1.5 μg of GFP, while Rev1pGFP in equivalent conditions produced around 3 μg (Figure 2b). This quantification was correlated with the existence of a *gfp* gene single copy and the lower fluorescence detected in the Tn*7‐gfp* vs. pBBR‐*gfp* mutant strains.

### 3.3 Rev1::*gfp* maintained the residual virulence and efficacy of the reference Rev1 vaccine strain in mice

**Figure 3:**
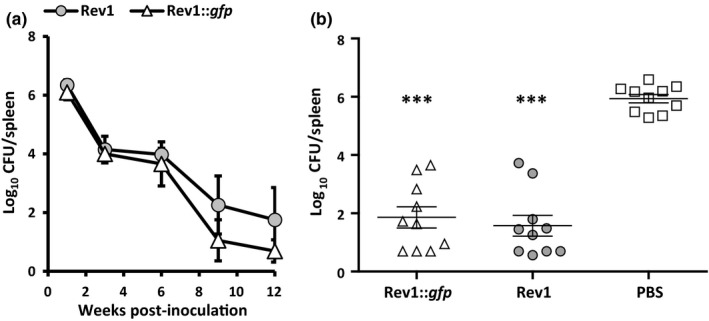
Rev1::*gfp* showed residual virulence and vaccine efficacy against a *Brucella melitensis* challenge similar to those of the parental Rev1 strain. (a) *Brucella* growth curves determined at 1, 3, 6, 9 and 12 weeks post‐inoculation in spleens of BALB/c mice inoculated IP with 1×10^6^ CFU of Rev1::*gfp* or Rev1 reference strains. Symbols represent the mean ± *SD* (*n* = 5) of individual log_10_ CFU/spleen at each selected interval. (b) Vaccine efficacy was determined in BALB/c mice (*n* = 10) vaccinated SC with 2 × 10^5^ CFU of Rev1::*gfp* or Rev1 reference‐type (vaccine control) or inoculated with 0.1 ml of PBS (unvaccinated control). All mice were challenged IP 4 weeks later with 1 × 10^4^ CFU/mouse of *B. melitensis* H38::Gm, and killed 2 weeks thereafter for assessing the number of H38::Gm CFU/spleen. Symbols represent the individual log_10_ CFU/spleen and lines represent the mean ± *SE* (*n* = 5) of each group. ***Fisher's PLSD test: *p* ≤ 0.001 vs. unvaccinated mice (PBS control)

As shown in Figure 3a, Rev1::*gfp* reproduced the general pattern of attenuation of the reference Rev1 strain. Accordingly, complete clearance of Rev1::*gfp* was observed between 9 and 12 weeks post‐vaccination. Likewise, Rev1::*gfp* conferred a protective efficacy against challenge with virulent *B. melitensis* H38::Gm similar to that induced by the reference Rev1 strain (Figure 3b).

### 3.4 Recombinant GFP was immunogenic and highly stable to temperature and UV radiation

The stability of recombinant GFP and GFP‐GST used for animal immunizations and for ELISA‐GFP was assessed under stressing conditions. A total loss of fluorescence was observed after 1 week of incubation at 60°C, and it was accompanied by a significant loss of antigenicity (Supporting information Figure S1). Full antigenicity and a slight loss of fluorescence were observed after a 9‐week incubation period at 37 and 44°C. Storage at 4°C for at least 5 months did not affect the fluorescence or antigenicity of GFP. Both, GFP and GFP‐GST were stable after 90 hr UV radiation (Supporting information Figure S1).

### 3.5 Anti‐GFP antibody response in mice

**Figure 4:**
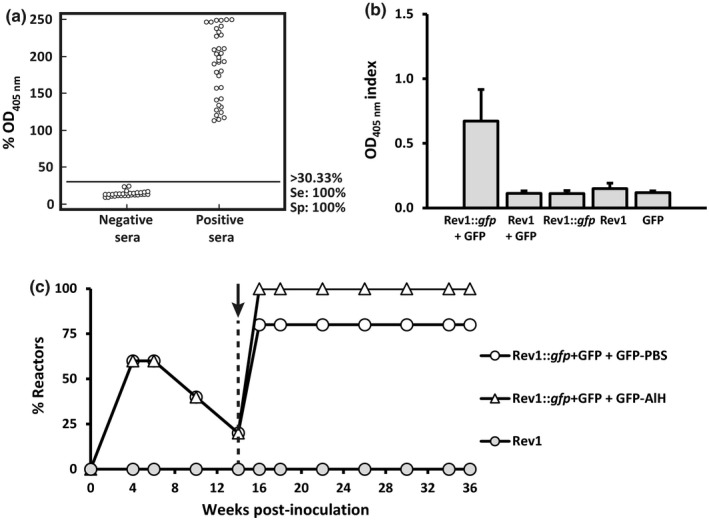
Serological response studies in BALB/c mice. (a) Determination of the optimal ELISA‐GFP cut‐off (≥30.33% OD_405 nm_) allowing a suitable discrimination between GFP‐negative (*n* = 30) and GFP‐positive (*n* = 36) mice sera. ELISA values are expressed as % OD_405nm_ = [(A/C_positive_) × 100], where A and C_positive_ are the raw data of absorbance obtained for the sample tested and the positive control, respectively. (b) ELISA‐GFP results obtained in BALB/c mice (*n* = 5) at 6 weeks after IP inoculation with 1 × 10^6^ CFU/mouse of Rev1::*gfp* alone (Rev1::*gfp)* or mixed with 20 μg/mouse recombinant GFP (Rev1::*gfp* + GFP). As controls, additional groups of mice (*n* = 5) inoculated IP with 1 × 10^6^ CFU/mouse of Rev1 alone (Rev1) or mixed with 20 μg/mouse recombinant GFP (Rev1 + GFP) or inoculated with 20 μg/mouse of recombinant GFP alone (GFP) were included. Values represent the mean ± *SE* (*n *= 5) of OD_405 nm_ index = (A–C_negative_), where A and C_negative_ are the raw data of absorbance obtained for the sample tested and the negative control respectively. (c) Percentage of mice (*n* = 5) positive in ELISA‐GFP after IP vaccination with 1 × 10^6^ CFU/mouse of Rev1::*gfp* mixed with 20 μg/mouse of recombinant GFP and boosted 14 weeks later (dashed vertical line) with recombinant GFP either in PBS (circles) or in 6 μg/mouse aluminum hydroxide (AlH) (triangles). Duplicate serum samples were tested at the selected intervals (abscissa axis) and the percentage of GFP‐positive mice at each point‐time assessed using a cut‐off ≥30.33% OD_405 nm_

The optimal ELISA‐GFP cut‐off (allowing a proper differentiation of GFP‐positive and GFP‐negative sera) was ≥30.33% OD_405 nm_ (Figure 4a). While a single dose Rev1::*gfp* vaccination did not induce detectable antibodies against GFP (Figure 4b), a significant anti‐GFP response was produced when mice were vaccinated simultaneously with Rev1::*gfp* and GFP (Figure 4b). This effect was not due exclusively to the exogenous GFP, since both the GFP given alone or inoculated simultaneously with the Rev1 standard vaccine did not induce detectable anti‐GFP antibodies (Figure 4b). Thus, the constitutive expression of *gfp* together a co‐immunization with GFP seems to be required to induce a detectable ELISA‐GFP antibody response. In fact, an important proportion (about 60%) of mice vaccinated with Rev1::*gfp* inoculated simultaneously with GFP (group Rev1::*gfp*+GFP, Figure 4c) resulted positive in ELISA‐GFP during the first 6 weeks post‐vaccination, declining thereafter. However, after boosting these mice with recombinant GFP either in PBS or AlH adjuvant, a significant increase of GFP response was evident at 2 weeks post‐booster and persisted for at least 22 weeks post‐booster (the end of the observational period; Figure 4c).

### 3.6 Safety and serological response to Rev1::*gfp* combined with recombinant GFP in sheep

All Rev1::*gfp* vaccination combinations resulted innocuous for the lambs. Only mild local abscesses were caused by Rev1::*gfp* in some lambs, that resolved spontaneously few weeks after vaccination, as reported to Rev1 (Barrio et al., 2009).

**Figure 5:**
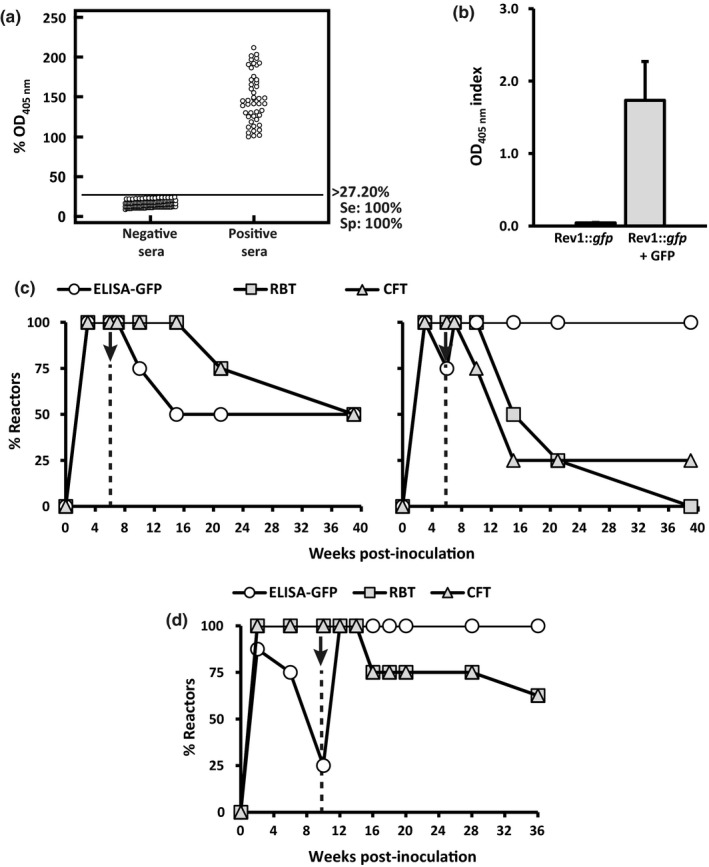
Serological responses in Rev1::*gfp* vaccinated lambs. (a) A cut‐off of 27.20% OD_405 nm_ or higher allowed a suitable discrimination of the GFP‐negative (*n* = 124) and GFP‐positive (*n* = 47) control sera. (b) Anti‐GFP response assessed 3 weeks after SC vaccination with 1–2 × 10^9^ CFU of Rev1::*gfp* given alone (*n* = 6) or mixed with 100 μg GFP/lamb (*n* = 8). Values represent the mean ± SE of OD_405 nm_ index. (c) percentage of reactors in ELISA‐GFP (circles), RBT (squares) and CFT (triangles) for 39 weeks after SC vaccination with Rev1::*gfp *+ GFP and a further boost 6 weeks later with GFP in PBS (*n *= 4; left panel) or in adjuvant (*n* = 4; right panel). (d) Evolution of the percentage of reactors (*n* = 8) in ELISA‐GFP (circles), RBT (squares) and CFT (triangles) in lambs (*n* = 8) immunized simultaneously with Rev1::*gfp* and GFP, and further boosted 10 weeks thereafter with GFP in AlH adjuvant

Regarding the ELISA‐GFP validation, a cut‐off of 27.20% OD_405 nm_ or higher allowed a suitable discrimination of the 47 GFP‐positive and the 124 GFP‐negative sheep sera used as controls (Figure 5a). Similarly to mice, lambs vaccinated with Rev1::*gfp* alone (*n* = 6) did not develop a significant anti‐GFP response, while the combined administration of this vaccine with recombinant GFP (*n* = 8) did (Figure 5b). Also, a further booster with GFP induced positive anti‐GFP reactions in all lambs. This anti‐GFP response was somewhat more intense when the GFP was given in IFA adjuvant instead of PBS (Figure 5c). As it can be seen in Figure 5d, in the second experiment, all lambs vaccinated with Rev1::*gfp*+GFP resulted positive in RBT and CFT after vaccination, and this anti S‐LPS response remained high along the whole observational period. Interestingly, all Rev1::*gfp*+GFP vaccinated animals boosted 10 weeks thereafter with GFP in AlH adjuvant showed a very high and durable anti‐GFP response that was maintained up to the end of the experiment (Figure 5d). Thus, this vaccination strategy allowed the identification of all vaccinated animals in a S‐LPS positive background.

## 4 DISCUSSION

Once sheep and goat brucellosis have been controlled by mass vaccination, eradication can be feasible combining vaccination and further testing and culling of seropositive animals (Blasco & Molina‐Flores, 2011). The live‐attenuated *B. melitensis* Rev1 is the only available effective vaccine that, when combined with appropriate testing and culling strategy, has allowed the eradication of small ruminant brucellosis in some countries (Blasco et al., 2016). However, Rev1 induces an anti S‐LPS response in vaccinated animals that makes eradication difficult. Thus, the generation of anti‐*Brucella* DIVA vaccines that could facilitate implementing eradication programs in small ruminants has been a recurrent topic of research through years (Blasco et al., 2016; Yang et al., 2013) and can be summarized in two approaches: (a) the removal of antigens of diagnostic value in field *Brucella* infections such as outer membrane and periplasmic proteins (Cloeckaert et al., 2004) or the S‐LPS (Godfroid et al., 2000; González et al., 2008) and (b) the genetic engineering approach used here, modifying the Rev1 vaccine by inclusion of foreign (xenogenic) antigens. Rev1 strains lacking protein antigens of diagnostic significance were generated in the past (Cloeckaert et al., 2004) and tested in sheep (Jacques et al., 2007). The deletion of *bp26* gene in the Rev 1 vaccine strain did not alter its biological properties but the diagnostic performance of the BP26 protein was only moderate due either to the low sensitivity of the BP26‐ELISA associated test or because *Brucella* infected sheep did not generate detectable amounts of anti‐BP26 antibodies (Grilló et al., 2009; Jacques et al., 2007). Other studies have been focused on the inactivation or deletion of genes involved in *B. melitensis* S‐LPS biosynthesis (Godfroid et al., 2000; González et al., 2008). This strategy may result in excessively attenuated candidates, which limit their protective efficacy (Barrio et al., 2009; González et al., 2008). Moreover, the rough mutants generated antibodies interfering in some S‐LPS tests used for diagnosing *B. melitensis* infection in small ruminants such as the iELISA, the cELISA and FPA (Moriyón et al., 2004; Barrio et al., 2009). In addition to our research by *gfp* stable insertion in the *Brucella* chromosome, vaccine prototypes using non‐integrative plasmids carrying heterologous genes have been constructed (Comerci et al., 1998; Pollevick et al., 1991) but it requires associated diagnostic tests allowing a suitable identification of vaccinated animals in infection contexts in which anti S‐LPS antibodies will coexist. The ELISA‐GFP developed and validated in our study fulfilled this requisite both in mice (Figure 4) and the target species (Figure 5).

A critical characteristic of any new tagged *Brucella* vaccine strain is that it should be easily and unequivocally differentiated from its parental counterpart, and also from other brucellae. Following above approach (b) we constructed Rev1::*gfp*, a Rev1 strain carrying a mini‐Tn*7*‐*gfp* inserted stably in the intergenic chromosomal region between *glmS‐recG* genes. While maintaining the microbiological characteristics of the classical Rev1 vaccine strain, Rev1::*gfp* can be unambiguously differentiated from Rev1 and other brucellae by both direct visualization under UV illumination and the associated multiplex PCR‐GFP developed. Moreover, any tagged vaccine for small ruminant brucellosis would be useful only if its virulence pattern and protection conferred are, at least, equivalent to that of the classical Rev1 vaccine. As seen in Figure 3, Rev1::*gfp* kept the virulence behaviour of Rev1 and conferred a similar level of protection against a virulent challenge in mice.

Rev1::*gfp* emitted less fluorescence and synthetized lower amounts of GFP than the Rev1pGFP prototype, which carried a high number of *gfp* copies. The low expression of GFP was consistent with the low level of anti‐GFP antibodies induced in both mice (Figure 4) and lambs (Figure 5) vaccinated with the Rev1::*gfp* given alone. Thus, Rev1::*gfp* required the co‐administration of exogenous recombinant GFP to increase the level of anti‐GFP antibodies. Preliminary studies were conducted in mice to establish the vaccine combination resulting in the higher anti‐GFP antibody response in lambs. Studies in mice proved that the recombinant GFP given alone lacks immunogenicity, as it has been reported previously (Fric, Marek, Hruskova, Holan, & Forstova, 2008); and that the co‐administration of Rev1::*gfp* with recombinant GFP increased the anti‐GFP response but not in a persistent way (Figure 4). By contrast, a further adjuvant‐GFP booster induced a high and durable anti‐GFP antibody response (Figure 4). Altogether, mice experiments proved that an exogenous GFP booster was essential for a suitable anti‐GFP response after Rev1::*gfp* vaccination, but also that the endogenous expression of GFP induced during Rev1::*gfp* replication in vivo could be required also to generate this durable anti‐GFP response. This requirement has been reported in experiments using other live‐attenuated bacteria as vectors of xenogenic antigens (Kotton & Hohmann, 2004). Finally, in contrast to that reported in other Gram negative bacteria (Gupta & Chaphalkar, 2015), the *B. melitensis* Rev1 S‐LPS does not act as an adjuvant for GFP protein.

As in mice, Rev1::*gfp* needed the simultaneous administration of GFP to induce anti‐GFP antibodies in lambs (Figure 5b). Moreover, an adjuvant‐GFP booster induced an intense and long‐lasting anti‐GFP antibody response in all vaccinated lambs (Figure 5c and d). As expected ideally, all Rev1::*gfp* vaccinated animals that resulted positive in the RBT and CFT standard S‐LPS tests were also showing anti‐GFP antibodies, which remained along the whole observational period, even in the animals that resulted negative in S‐LPS tests (Figure 5d). This proves the usefulness of the GFP tagging to identify the Rev1::*gfp* vaccinated animals in a context in which the anti‐S‐LPS antibodies are prevalent.

The availability of a GFP tagged Rev1 strain able to express GFP more efficiently than our Rev1::*gfp* construct could avoid the need of using recombinant GFP for both co‐vaccination and booster, thus reducing the costs of the complex vaccination procedure. As mentioned above, the Rev1pGFP synthetized significantly higher GFP amounts than the Rev1::*gfp* construct and retained the biological properties of Rev1 (not shown) but, unfortunately, this prototype was unable to retain the non‐integrative plasmid. To improve the tagging of Rev1 we are conducting currently different approaches to insert either one mini‐Tn*7*‐*gfp* carrying multiple *gfp* copies or multiple mini‐Tn*7* in *att*Tn*7* sites generated artificially, which have been tested successfully in other bacteria (Choi, Bourque, Morel, Groleau, & Míguez, 2006; Roos, Werner, & Loessner, 2015). However, an equilibrium between GFP expression and the maintenance of the vaccine properties should be taken into account to avoid over attenuated constructs resulting in poor protective efficacy. Also, we are currently applying the GFP tagging in alternative vaccine candidates generating less anti S‐LPS antibodies than Rev1 and/or lacking antigens of diagnostic significance in virulent *Brucella* strains. Combination of both approaches could be a valuable tool for the eradication of brucellosis in contexts in which vaccination is required.

## CONFLICT OF INTEREST

The authors declare that there are no conflicts of interests.

## AUTHORS CONTRIBUTIONS

A.Z.B. and B.S.R. have constructed the plasmids and the Rev1::*gfp* mutant, produced the GFP recombinant and performed the experiments of Rev1::*gfp* and GFP stability, mice assays, ELISA‐GFP validation and serology in mice and sheep; C.C.D. designed the GFP‐GST construction and PCR‐GFP; M.I. advised in the design of the mini‐Tn7‐*gfp* chromosomal insertion protocol; M.J.d.M., P.M.M. and J.M.B. carried out the vaccination and sampling of sheep; M.J.G. made the design and conceptualized the study; A.Z.B., B.S.R., J.M.B. and M.J.G. were involved in data acquisition and analysis, manuscript writing and proof reading revision. All authors gave corrections to the manuscript and final approval for publication.
