# Supplementary material for: GFP tagging of Brucella melitensis Rev1 allows the identification of vaccinated sheep
Source: Transbound Emerg Dis. 2018 Nov 26;66(1):505–16. doi: 10.1111/tbed.13053 (PMC7379934; doi:10.1111/tbed.13053)
Supplement: Supplementary file 2 [file TBED-66-505-s002.docx]

**Table S1.** Analysis *in silico* of *glmS* in the chromosome II of *Brucella* spp.

| Strain | Annotation | *glmS* gene position | % of nucleotide identity vs. 16M |
| --- | --- | --- | --- |
| *B. melitensis* 16M | NC_003318 | 491663...493486 | - |
| *B. abortus* 2308 | NC_007624 | 651243...653066 | 99 |
| *B. abortus* S19 | CP000888.1 | 650458...652281 | 99 |
| *B. suis* 1330 | NC_004311 | 563998...565821 | 99 |
| *B. ovis* ATCC 25840 | NC_009504 | 565393...567216 | 99 |
| *B. canis* ATCC 23365 | NC_010104 | 563983...565806 | 99 |
| *B. microti* CCM4915 | NC_013118 | 565429...567252 | 99 |
| *B. pinnipedialis* B2/94 | NC_015858 | 618859...620682 | 99 |
| *B. ceti* TE10759-12 | CP006897.1 | 707921...709744 | 99 |
